# Silica Nanoparticles Enhance the Disease Resistance of Ginger to Rhizome Rot during Postharvest Storage

**DOI:** 10.3390/nano12091418

**Published:** 2022-04-21

**Authors:** Jie Zhou, Xuli Liu, Chong Sun, Gang Li, Peihua Yang, Qie Jia, Xiaodong Cai, Yongxing Zhu, Junliang Yin, Yiqing Liu

**Affiliations:** 1 Spice Crops Research Institute, College of Horticulture and Gardening, Yangtze University, Jingzhou 434025, China; zj188719@163.com (J.Z.); liuxuli33@163.com (X.L.); zbgqsc1987@163.com (C.S.); lg13733590933@163.com (G.L.); yph919701@163.com (P.Y.); jiaqie020@163.com (Q.J.); caixiao.dong@163.com (X.C.); 2 Special Plants Institute, College of Landscape Architecture and Life Science, Chongqing University of Arts and Sciences, Chongqing 402160, China

**Keywords:** silica nanoparticles, postharvest decay, fungal disease, *Zingiber officinale*

## Abstract

Silica nanoparticles (SiNPs) offer an ecofriendly and environmentally safe alternative for plant disease management. However, the mechanisms of SiNPs-induced disease resistance are largely unknown. This research evaluated the application of SiNPs in controlling the postharvest decay of ginger rhizomes inoculated with *Fusarium solani*. In vitro study showed that SiNP had little inhibitory effect on mycelial growth and spore germination of *F. solani* and did not significantly change mycelium’s MDA content and SDH activity. In vivo analysis indicated that SiNPs decreased the degree of decay around the wounds and decreased the accumulation of H_2_O_2_ after long-term pathogenic infection through potentiating the activities of antioxidant enzymes such as SOD, APX, PPO, and CAT. SiNP150 increased the CHI, PAL, and GLU activity at the onset of the experiment. Moreover, SiNP150 treatment increased total phenolics contents by 1.3, 1.5, and 1.2-times after 3, 5, and 7 days of treatment, and increased total flavonoids content throughout the experiment by 9.3%, 62.4%, 26.9%, 12.8%, and 60.8%, respectively. Furthermore, the expression of selected phenylpropanoid pathway-related genes was generally enhanced by SiNPs when subjected to *F. solani* inoculation. Together, SiNPs can effectively reduce the fungal disease of ginger rhizome through both physical and biochemical defense mechanisms.

## 1. Introduction

Ginger (*Zingiber officinale* Roscoe), one of the most economically important vegetables in the Zingiberaceae family, has been widely planted worldwide [1]. It is used as food, spice, flavoring agent and herbal remedy owing to its beneficial characteristics relating to its aroma, pungency, nutrients, and medicinal properties [2]. Ginger suffers from dehydration, sprouting, and decay caused by pathogens during storage after 3 to 4 weeks of harvesting [3]. Significant losses in harvested ginger can be directly attributed to decay fungi. Soft rot (rhizome rot), generally caused by *Fusarium solani*, is one of the major constraints in the production of ginger both in the field and during storage [4]. It not only causes rhizome rot in ginger at different growth stages but also threatens the postharvest storability of ginger. Thus, there is an urgent need to reduce the fungal pathogen-induced decay and extend the postharvest life of ginger.

The application of chemical fungicides is considered an effective method to control the soft rot of ginger; nevertheless, the extensive use of fungicides poses a serious risk to environmental and human health. Disease-management tools, which can serve as alternatives to conventional synthetic chemical fungicides for postharvest disease control, are being actively investigated in many horticultural plants [5]. Several nanoparticles, such as silver nanoparticles, TiO_2_ nanoparticles, and ZnO nanoparticles, have been investigated in postharvest management and developed to control disease in citrus, grape, banana, apple, mango, peach, and nectarine [6]. Silicon (Si) is generally regarded as a safe substance (GRAS) and an effective elicitor to activate plant defenses [7]. Recently, Silica nanoparticles (SiNPs; food additive E551) were reported as a novel Si source [8] that can be used to improve plant resistance to salt, drought, and disease [9] and are considered more efficient than their bulk particles owing to their small size and high surface area and reactivity. Moreover, SiNPs exhibit potent antibacterial properties against a variety of plant diseases through the formation of physical barriers (deposition and polymerization of Si below the cuticle) or the induction of biochemical reactions (production of secondary metabolites). They can also modulate plant antioxidant activity. For example, as mentioned in a recent report, SiNPs have the potential to induce local and systemic disease resistance in *Arabidopsis thaliana* against the bacterial pathogen *Pseudomonas syringae* [9]. Thus, exogenous SiNPs have significant prospects as an inexpensive, highly efficient, and sustainable alternative in environmentally friendly disease management.

Initial studies found that SiNPs may induce stress tolerance similar to conventional Si products, but a clear mechanistic understanding of the underlying processes is still lacking. Moreover, as a food additive (SiNPs), there is little literature available on the role of SiNPs in the postharvest handling and storage of vegetables. Therefore, this study explores the mechanism by which SiNPs induce the postharvest resistance of ginger infected with *F. solani*. We first determined the effect of SiNPs on the postharvest disease resistance of ginger, following which the initiation mechanism of defense responses was elucidated through a comprehensive analysis of antioxidant enzymes, disease resistance-related enzyme activities, secondary metabolites, and reactive oxygen species (ROS), as well as key genes in the lignin and flavonoid synthesis processes. This research will provide a foundation for using inexpensive and highly efficient SiNPs in postharvest disease management.

## 2. Materials and Methods

### 2.1. Plant Materials

Healthy ginger rhizomes (*Z. officinale Ros.* cv. fengtou) were harvested at the Yangtze University ginger planting base (E:112.026207, N:30.361273) in November (180 days after planting) and immediately taken to the laboratory within 2 h. Ginger with uniform size, identical color, without signs of pests or diseases, and being free of any mechanical damages or fungal decays were selected to perform the following experiments.

### 2.2. Fungal Pathogen

The pathogenic fungi of *Fusarium solani* were isolated from the rhizomes of rotten ginger and identified by morphology and sequence of the internal transcribed spacer 1 (ITS1 primer: 5′-GCTCAGCGGCTTCCTATTG-3′) and internal transcribed spacer 4 (ITS4 primer: 5′-CGGGGTATTCATCATTCACTTCA-3′) rDNA region. Then *F. solani* was cultured onto a potato dextrose agar (PDA) plate at 28 °C. After 7 days of culture, the surface of the mycelium was gently washed with 0.05% Tween-80 and filtered with a double-layer gauze to obtain the spore suspension. A spore suspension was adjusted to 1 × 10^8^ spores mL^−1^ with a hemocytometer before use.

### 2.3. Effect of SiNPs on F. solani In Vitro

Silica nanoparticles (SiNPs) are an E551 food additive purchased from Sigma-Aldrich (Lot 637238, the purity is 99.5%, and particle size is 10–20 nm). The SiNP (50 mg L^−1^, 100 mg L^−^^1^, and 150 mg L^−1^) was suspended in water by sonicating the silica bundles via an ultrasonicator at 10 MHz for ∼40 min resulting in a partially homogeneous solution.

#### 2.3.1. Determination of Mycelial Growth

The inhibitory effect of SiNPs on the mycelial growth of *F. solani* was determined by the agar dilution method [10]. Briefly, sterile water, 0.05% tween-80, SiNP50 (50 mg L^−1^), SiNP100 (100 mg L^−1^), and SiNP150 (150 mg L^−1^) were diluted into a melting PDA medium respectively and poured into 60 mm-diameter petri dishes. The 6 mm-diameter mycelial disks taken from a 5-day-old culture of *F. solani* were placed in the center of each petri dish and incubated at 28 °C. Mycelial growth was then surveyed by measuring the diameter of the colonies using the cross-bonded method after 5 days. Then, sterile water-washed mycelia were collected at 5 days and stored at −80 °C until use.

#### 2.3.2. Determination of Spore Germination

A spore suspension was prepared as described before. Spore suspension (200 µL) was added into the PDA medium (containing sterile water, 0.05% tween-80, and SiNP150, respectively). The germination of the spore was evaluated with the aid of an optical microscope (DM500, Leica, Wetzlar, Germany). Each treatment randomly selected three visual fields to calculate the number of germinating spores. The experiment was conducted twice.

#### 2.3.3. Determination of Malondialdehyde (MDA) Content and Succinate Dehydrogenase (SDH) of *F. solani*

About 0.5 g of mycelium was ground using 1 mL phosphate buffer (0.1 M, pH 7.5). Then the mixture was treated with an ultrasonic cell crusher (JY92IIDN, Ningbo Xinzhi Biotechnology Co., Ltd., Ningbo, China) for 5 min (200 W, interval 2 s) to break the cell. After centrifuging at 4000× *g* for 10 min at 4 °C, the supernatant was collected and used as a crude enzyme solution for further analysis.

MDA content was determined according to Dhindsa et al. [11]. About 2 g mycelium sample was homogenized with 2 mL of 10% cold thiobarbituric acid (TCA) and then incubated in an ice bath for 10 min. After centrifugation, the 2 mL supernatant was collected and mixed with 2 mL thiobarbituric acid, and this mixture was boiled in a water bath for 20 min. The absorbance was measured at 450, 532, and 600 nm. The MDA content was calculated using the following formula and expressed as nmol g^−1^ of mycelium fresh weight basis: MDA content (nmol g^−1^) = 6.45 × (OD_532_ − OD_600_) − 0.56 × OD_450_.

Succinate dehydrogenase activity was assayed using an assay kit (Nanjing Jiancheng Bioengineering Institute, China) by measuring the absorbance at 600 nm.

### 2.4. SiNPs Dioxide Treatment of Postharvest Ginger Rhizomes

All ginger rhizomes were uniformly wounded with a sterilized borer (6 mm deep × 6 mm wide) and randomly divided into four groups. Ginger rhizomes were treated as follows: (1) Control (CK), ginger rhizomes immersed in sterile-distilled water for 10 min at room temperature. After being air-dried, ginger rhizomes were inoculated with 100 µL of sterile water into the wound. (2) SiNP150, ginger rhizomes immersed into 150 mg L^−1^ SiNPs for 10 min at room temperature. After air-dried, ginger rhizomes were inoculated with 100 µL of sterile water into the wound. (3) *F. solani*, ginger rhizomes immersed in sterile-distilled water for 10 min at room temperature. After being air-dried, ginger rhizomes were inoculated with 100 µL of the *F. solani* spore suspension into the wound. (4) SiNP150 + *F. solani*, ginger rhizomes immersed in 150 mg L^−1^ SiNPs for 10 min at room temperature. After being air-dried, ginger rhizomes were inoculated with 100 µL of the *F. solani* spore suspension into the wound. All treated ginger were separately incubated in a plastic box covered with preservative film at 28 °C and 90 ± 5% relative humidity for 7 d. A 15–20 mm sample annulus around the wound was collected at 0.5, 1, 3, 5, and 7 days after treatment. Each sample was frozen in liquid nitrogen immediately and stored at −80 °C until analysis.

### 2.5. Scanning Electron Microscope (SEM) Analysis

For SEM analysis, tissue sections of 10 mm × 10 mm × 2 mm were fixed in 2.5% glutaraldehyde solution and dehydrated in different concentrations of anhydrous ethanol (30%, 50%, 70%, 80%, 90%, 95% and 100%) [12]. Then the sample was dried thoroughly for 2 h in a Critical Point Dryer (Rockville, Maryland, USA), sputter-coated with gold at 5 mA and 1.5 kV using a coater (Ion Sputter JFC-1100, Tokyo, Japan), and then observed using a JSM-7100F Scanning Electron Microscope (Tokyo, Japan).

### 2.6. Determination of H_2_O_2_, O_2_^−^ and MDA

#### 2.6.1. DAB and NBT Histochemical Staining and Determination of H_2_O_2_ and O_2_^−^ Content

The DAB (diaminobenzidine) and NBT (Nitroblue tetrazolium) were used for histochemical staining of hydrogen peroxide (H_2_O_2_) and superoxide anion (O_2_^−^), respectively [13]. Briefly, for histochemical staining of H_2_O_2_, ginger from different treatments for 7 days was cut into thin slices from the wound and placed in a solution of DAB (1 mg mL^−1^, pH 5.0, dissolved in 10 mM Tris-acetate) and vacuum pumping for 5 min, then the samples were placed in the dark for 3 h at room temperature until brown spots appeared. In order to detect O_2_^−^, the ginger slices were soaked in a 0.1% solution of NBT (dissolved in 10 mM K-phosphate buffer, pH 6.4). Then samples were vacuum-infiltrated for 10 min and illuminated until the appearance of dark blue spots and then photographed. All samples collected after 7 days were used to determine the content of H_2_O_2_ and O_2_^−^ according to Kumar et al. [14].

#### 2.6.2. Determination of Malondialdehyde (MDA) Content

MDA content was determined as described in Section 2.3.3.

### 2.7. Enzyme Assays

About 1.0 g of fresh tissue was ground with 9.0 mL sodium phosphate buffer (50 mM, pH 7.0), and collected the homogenate centrifuged at 9661 *g* for 20 min at 4 °C. The supernatant was used as an enzyme source to measure peroxidase (POD, EC 1.11.1.7), superoxide dismutase (SOD, EC 1.15.1.1), catalase (CAT, EC 1.11.1.6), ascorbate peroxidase (APX, EC 1.11.1.11), phenylalanine ammonia-lyase (PAL, EC 4.3.1.5), polyphenol oxidase (PPO, EC.1.10.3.1), chitinase (CHI, EC 3.2.1.14) and β-1,3-glucosidase (GLU, EC 3.2.1.73) activities. The protein content in the crude enzyme extract was measured according to Bradford [15]. The specific activity of all the enzymes was expressed as units per milligram protein (U mg^−1^ protein).

POD activity was measured according to the method of Wang et al. [16]. We mixed 3.0 mL guaiacol solution (25 mM) with 0.5 mL of enzyme extract, and then 200 µL of a 0.5 mol L^−1^ H_2_O_2_ solution was added and mixed quickly to start the reaction. POD activity was defined as the amount of enzyme that caused an increase in absorbance of 0.01 per min at 470 nm.

The reaction system (3.0 mL) for SOD activity contained a 65 mmol L^−1^ sodium phosphate buffer (pH 7.8), 13 mmol L^−1^ methionine, 75 μmol L^−1^ nitro blue tetrazole (NBT), 10 μmol L^−1^ EDTA, 2 μmol L^−1^ riboflavin and 0.1 mL of an enzyme extract. After mixing, we placed the control tube in the dark and the determination tube in the light for 15 min; then, the absorbance was measured at 560 nm [17].

CAT activity measuring refers to the method of Zhu et al. [18]. Specifically, about 2.9 mL of 20 mmol L^−1^ H_2_O_2_ solutions were mixed with 0.1 mL of enzyme extract. Then the absorbance was measured at 240 nm every 30 s. One unit was defined as a change of 0.01 per min.

Reaction mixtures for APX activity determination contained 2.4 mL sodium phosphate buffer (50 mM, pH 7.5), 0.2 mL of 2 mmol L^−1^ H_2_O_2_ and 0.4 mL of supernatant. The APX activity was estimated by the decrease in absorbance at 290 nm [19].

For PAL, the reaction system contained 1.0 mL of 0.02 mol L^−1^ phenylalanine, 2.0 mL of boric acid buffer (pH 7.8), 1.0 mL of an enzyme solution; we shook this well, and then placed it in a 30 °C water bath for 60 min. After that, 0.2 mL of 6 mol L^−1^ HCl was added to terminate the reaction. The absorbance of the reaction solution was detected at 290 nm [20].

CHI and GLU activity was determined using Enzyme Activity Kit (Solarbio, China, BC0820 and BC0360) following the instructions, and the OD value was measured at 585 nm and 540 nm, respectively.

### 2.8. Determination of Total Phenolics, Total Flavonoid and Lignin Contents

Total phenolics and flavonoid contents were measured according to the Toor and Savege method [21]. Briefly, 0.25 g freeze-dried ginger powder was dissolved into 8 mL distilled water and sonicated for 60 min to dissolve the residue completely. Then, the mixture was centrifuged at 9661 *g* for 20 min. The 0.1 mL supernatant and 0.4 mL distilled water were added to 0.5 mL of 1 mol L^−1^ Folin-Ciocalteau reagent. After reaction for 5 min in the dark, 2.0 mL of 7.5% Na_2_CO_3_ solution was added to the mixture, and 1.0 mL distilled water was finally added and mixed well. The mixture was incubated at 40 °C for 30 min in the dark. The absorbance was measured at 765 nm. Total phenolic content was expressed as g of gallic acid per kg of sample dry weight basis.

Total flavonoid was determined according to Zhi et al. [22]. About 0.05 g of dried ginger powder was added to 1.0 mL of 60% ethanol and ultrasonically extracted at 60 °C for 30 min, and then centrifuged at 9661 *g* for 10 min to obtain supernatant. The supernatant and 15 μL 5% sodium nitrite solution were mixed and placed at a normal temperature reaction for 5 min. Then we added 15 μL Al(NO_3_)_3_ solution with 10% mass fraction, standing at normal temperature for 5 min, 120 μL of NaOH solution with 4% content, and 90 μL of ethanol solution with 60% volume fraction were finally added and mixed and bathed in water at 37 °C for 45 min. The absorbance was measured at 510 nm, and the result was expressed as rutin per kg of fruit dry weight basis.

Lignin was detected quantitatively using a lignin thioglycolic acid method [23]. About 3.0 g of ginger tissue was ground into homogenate with 5 mL of 95% ice ethanol. The homogenate was centrifuged at 9661 *g* for 10 min at 20 °C; the precipitate was rinsed three times with 95% alcohol, then the mixture was rinsed with ethanol and n-Hexane (Vethanol:VHexane, 1:2) three times and the samples were dried at 80 °C in the oven. Then the precipitate was added to 25% acetyl bromide (dissolved with glacial acetic acid) and incubated at 70 °C for 30 min; 1.0 mL of 2 mol L^−1^ NaOH was used to terminate the reaction. Then 0.1 mL of 7.5 mol L^−1^ hydroxylamine hydrochloride and 2.0 mL glacial acetic acid were added to the mixture and centrifuged at 30,000× *g* for 10 min at 20 °C. Finally, acetic acid was added to 20 μL supernatant to a final volume of 5 mL. Absorbance was measured at 280 nm.

### 2.9. Quantitative Real-Time PCR (qRT-PCR) Assays

*F. solani* and SiNP150 + *F. solani* treated ginger rhizomes were used to perform qRT-PCR analysis. Total RNA was prepared using the Trizol reagent (Invitrogen) in accordance with the manufacturer’s protocol. Total RNA (1 μg) was used for cDNA synthesis using the HiScript Reverse Transcriptase with gDNA Eraser (Vazyme, Nanjing, China). The qRT-PCR analysis was performed with CFX 96 Real-Time PCR system (Bio-Rad) using a Cham SYBR qPCR Master Mix (Vazyme, Nanjing, China). The primer sequences used for qPCR analysis are listed in Supplementary Table S1. The relative expression levels of target genes were calculated with the 2^−ΔΔCt^ formula. Each sample consisted of three biological replicates.

### 2.10. Statistical Analysis

All experiments were performed using a completely randomized design, with three biological replicates for each treatment level. The results are presented as the mean ± SD. All statistical analyses were performed by SPSS 19.0. The data were subjected one-way analysis of variance (ANOVA), and mean separation was performed by Duncan’s multiple range test. The differences at *p* < 0.05 were considered significant. Data are presented as the means ± standard deviations (SD).

## 3. Results

### 3.1. Effect of SiNPs Treatment on F. solani In Vitro

To be more aware of the physical and chemical properties of the nanoparticles being used in this study, we performed a scanning electron microscope (SEM), transmission electron microscope (TEM), and Fourier Transform infrared spectroscopy (FTIR) spectra analysis of silica nanoparticles (Figure S1). The morphology of SiNPs characterized using SEM (Figure S1A) and TEM (Figure S1B) analyses revealed almost spherical nanoparticles. The FTIR spectra displayed the broad peaks detected at 1105.33 (corresponding to the Si-O-Si) and 470.95 cm^−1^ (corresponding to the Si-O band) ranges (Figure S1C). Therefore, this study used SiNPs due to their high stability and purity.

**Figure 1:**
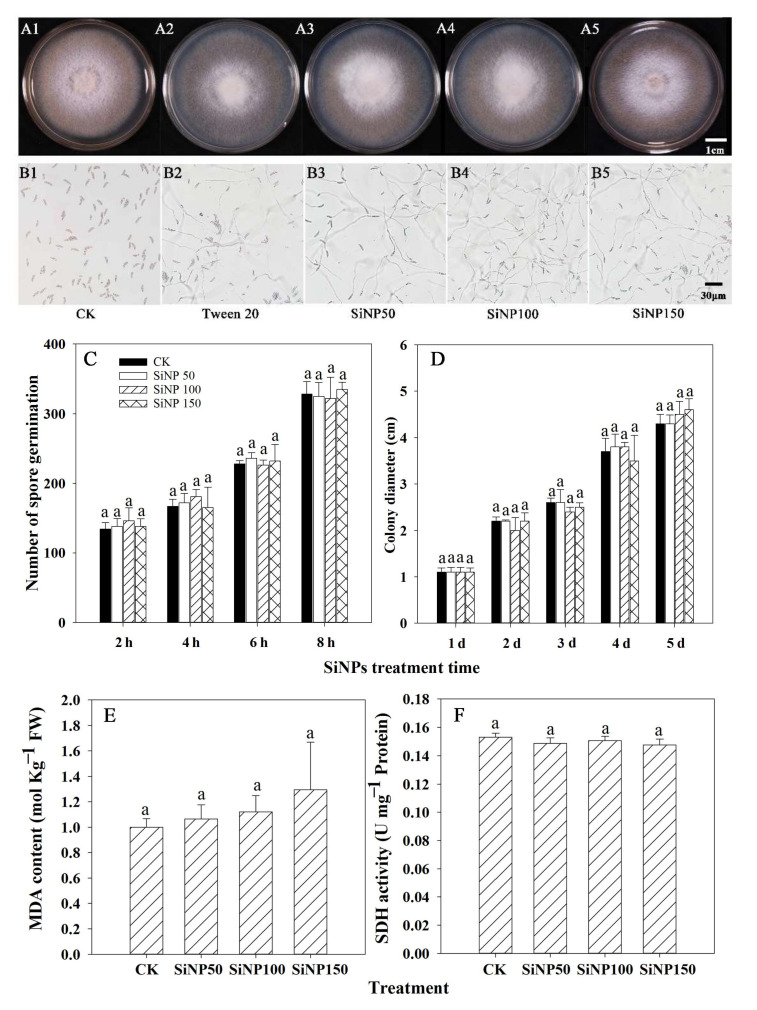
Effects of different concentrations of SiNPs on *F. solani* in vitro. (**A1**–**A5**) Effect of SiNPs treatment on mycelial growth after 7 days of treatment. (**B1**–**B5**) Effect of SiNPs treatment on spore germination after 6 h of treatment. Effect of SiNPs treatment on (**C**) spore germination, (**D**) colony diameter, (**E**) MDA content, and (**F**) SDH activity on *F. solani* after 7 days of treatment. Results represent the mean ± standard error deviation (SD). Different small letters in the figure show significant difference, the same letter indicates no difference (*p* < 0.05).

As can be seen from Figure 1A,B, different concentrations of SiNP treatment had little inhibitory effect on the colony diameter and spore germination compared with CK. However, the colony diameter and spore germination increased with the extension of inoculation time (Figure 1C,D).

To further determine the effects of SiNPs on *F. solani*, the malondialdehyde (MDA) content and SDH activity of the mycelia were measured. As shown in Figure 1E, compared with the control, SiNP50, SiNP100, and SiNP150 slightly increased the MDA contents, with the SiNP150-treated mycelia having the highest MDA content. Similarly, compared with the control, the SiNP50, SiNP100, and SiNP150 treatments did not affect SDH activity (Figure 1F).

### 3.2. Effect of SiNPs Treatment on Rhizome of Ginger Inoculated with F. solani

To further explore the potential mechanisms by which Si induces resistance to fungal pathogens in plants, the inhibitory effect of SiNPs against *F. solani* in vivo was measured. In preliminary experiments, we studied the effect of 50, 100, and 150 mg L^−1^ SiNPs on the postharvest disease resistance of ginger. The results showed that 150 mg L^−1^ (SiNP150) was the optimal concentration for controlling postharvest decay in ginger. Therefore, SiNP150 was selected and used for the following experiments.

**Figure 2:**
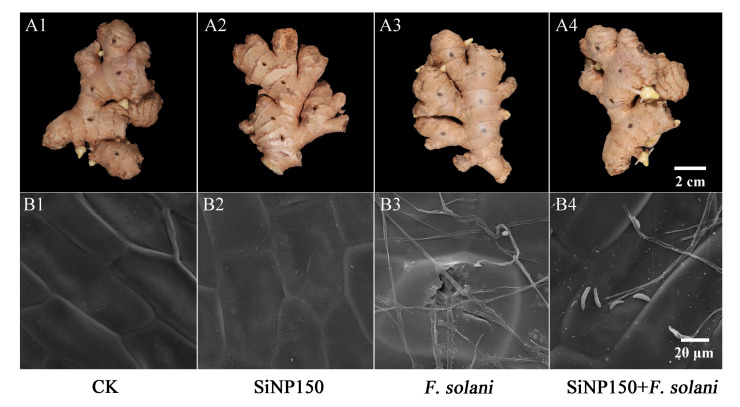
Effect of SiNP150 treatment on decay development and mycelia growth in ginger rhizome wounds caused by *F. solani* during postharvest storage. (**A1**–**A4**) The development of decay after inoculation for 7 days. (**B1**–**B4**) Scanning electron microscope observation of mycelia of *F. solani* treated with SiNP150 for 7 days.

As shown in Figure 2A, no difference was observed between the control and SiNP150 treatment after 7 days of inoculation (Figure 2A1,A2). In the *F. solani* treatment, obvious white hyphae grew superficially along the sample surface (Figure 2A3), whereas the hyphae were sparsely distributed around the wounds in the SiNP150-treated sample (Figure 2A4). SiNP150 treatment decreased the lesion diameter around the wounds compared to *F. solani* treatment alone (Figure 2A3,A4).

Scanning electron microscopy (SEM) observation of *F. solani* colonization was performed three days after inoculation. As shown in Figure 2B, obvious white Si deposition was observed in the SiNP150 and SiNP150 + *F. solani* treatment, and the epidermal cells of the control group and SiNP150 treatment were arranged neatly (Figure 2B1,B2). After *F. solani* treatment for 3 days, the hyphae of *F. solani* grow superficially along the sample surface. The hyphae of the inoculation group penetrated into the cell, whereas SiNP150 prevented hyphal penetration into the cells through the formation of white Si layers (Figure 2B3,B4).

### 3.3. Effect of SiNP150 on In Vivo Visualization and Content of Reactive Oxygen Species (ROS)

**Figure 3:**
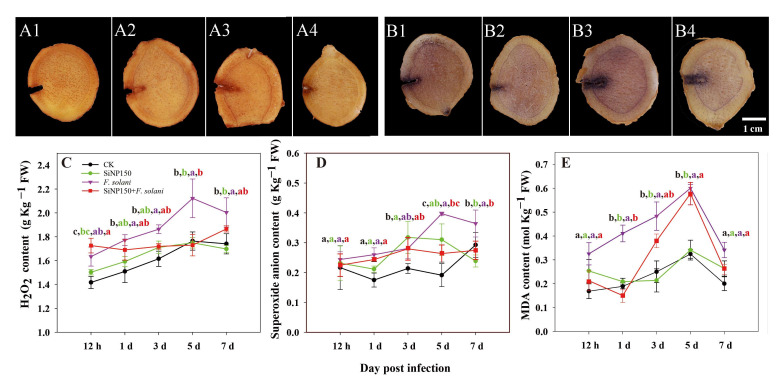
Effect of SiNPs and *F. solani* inoculation on the in vivo visualization of (**A1**–**A4**) O_2_^−^ and (**B1**–**B4**) H_2_O_2_, and the contents of (**C**) H_2_O_2_, (**D**) O_2_^−^ and (**E**) MDA of ginger rhizome. (**A1**) and (**B1**), CK; (**A2**) and (**B2**), SiNP150; (**A3**) and (**B3**), *F. solani*; (**A4**) and (**B4**), SiNP150 + *F. solani*. Results represent the mean ± standard deviation (SD) Different small letters in the figure show significant difference, the same letter indicates no difference (*p* < 0.05).

We further examined how SiNPs activate the biochemical defense response of ginger. For the visualization of H_2_O_2_ and O_2_^−^, histochemical analyses were performed at 7 days of treatment. Compared with CK and SiNP150, *F. solani* inoculation markedly increased the dye staining levels of H_2_O_2_ and O_2_^−^, while SiNP150 decreased H_2_O_2_ and O_2_^−^ staining compared with *F. solani* inoculation alone (Figure 3A,B).

Within 1–7 days of treatment, the contents of H_2_O_2_ and O_2_^−^ in each group showed an upward trend (Figure 3C,D). Compared with the control, *F. solani* inoculation increased the H_2_O_2_ and O_2_^−^ contents, whereas SiNP150 + *F. solani* inhibited these increases, especially after 3 days of treatment. Compared with *F. solani* treatment alone, SiNP150 + *F. solani* decreased the H_2_O_2_ content by 6.6%, 18.26%, and 14.29% after 3, 5, and 7 days of treatment. SiNP150 + *F. solani* decreased the O_2_^−^ content by 3.5%, 33.3%, and 24.8% after 3, 5, and 7 days of treatment, respectively.

The MDA content in each treatment group exhibited an upward trend during the entire storage process before 5 days. The SiNP150 treatment showed similar fluctuations as the control. The *F. solani* treatment increased the MDA content throughout the experiment, while SiNP150 + *F. solani* reduced and delayed MDA accumulation. It should be noted that the MDA content peaked at 5 days of treatment and then decreased in all samples, with *F. solani* being higher than SiNP150 + *F. solani*, SiNP150, and CK (Figure 3E).

### 3.4. Effects of the SiNPs Treatment on Antioxidant Enzyme Activities in Ginger Rhizome

**Figure 4:**
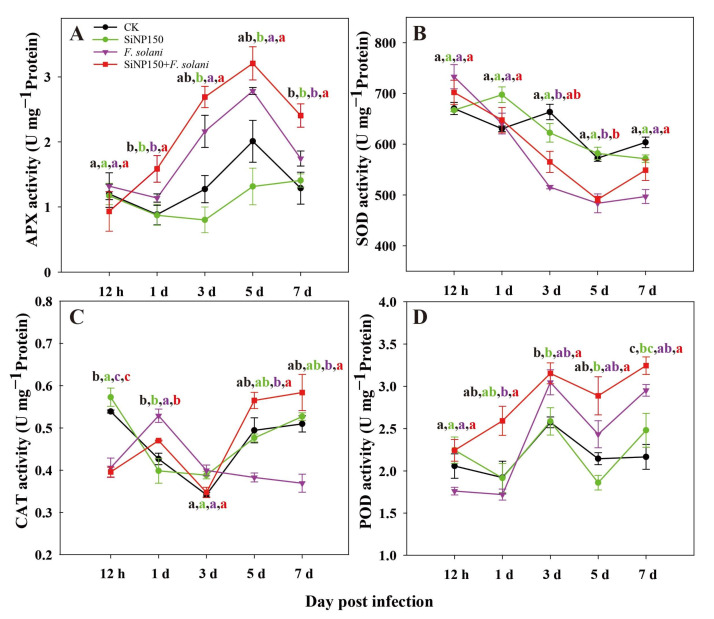
Effects of SiNPs and *F. solani* inoculation on the activities of (**A**) APX, (**B**) SOD, (**C**) CAT, and (**D**) POD activity in ginger rhizomes during postharvest storage. Results represent the mean ± standard deviation (SD). Different small letters in the figure show significant difference, the same letter indicates no difference (*p* < 0.05).

As can be seen in Figure 4, the peroxidase (POD), superoxide dismutase (SOD), catalase (CAT), and ascorbate peroxidase (APX) activities of the ginger rhizomes treated with SiNP150 generally showed similar fluctuating trends as the control.

Compared with the control, SiNP150 exerted little effect on the APX activity after 0.5, 1, and 7 days but decreased the APX activity after 3 and 5 days of treatment. Inoculation with *F. solani* induced the APX activity of the ginger rhizomes during the entire storage process as compared with the control and *F. solani* treatment. Compared with *F. solani* treatment alone, the SiNP150 + *F. solani* treatment increased the APX activity from 1 to 7 days, reaching a maximum of 3.25 on the 5th day.

The SOD activity of the ginger rhizomes generally showed a downward trend (Figure 4B). Compared with the control and SiNP150, *F. solani* and SiNP150 + *F. solani* treatment increased the SOD activity at 0.5 days of treatment but decreased it after 3 days of treatment. Compared with *F. solani* treatment alone, SiNP150 + *F. solani* treatment increased the SOD activity by 9.6%, 1.56%, and 13.8% after 3, 5, and 7 days of treatment (Figure 4B).

Before 3 days of treatment, the CAT activity of the SiNP150 + *F. solani* treatment showed similar trends to *F. solani* treatment alone but was lower than that of the *F. solani* treatment. After 5 and 7 days of treatment, the activities of CAT in the *F. solani* treatment surpassed that of the other three treatments. The CAT activity of the SiNP150 + *F. solani* treatment was significantly higher than the inoculation group and reached the maximum value of 0.58 (U mg^−1^ protein) on the 7th day, which was 1.6 times that of the *F. solani* treatment alone (Figure 4C).

Compared with the control and SiNP150, *F. solani* treatment alone decreased the POD activity before 1 day but increased it after 3 days of treatment. SiNP150 + *F. solani* increased the POD activity throughout the experiment compared with the other treatments, exhibiting the highest activity on the 7th day after inoculation followed by the 3rd day.

### 3.5. Effects of the SiNPs Treatment on Disease Resistance-Related Enzyme Activities in Ginger Rhizome

**Figure 5:**
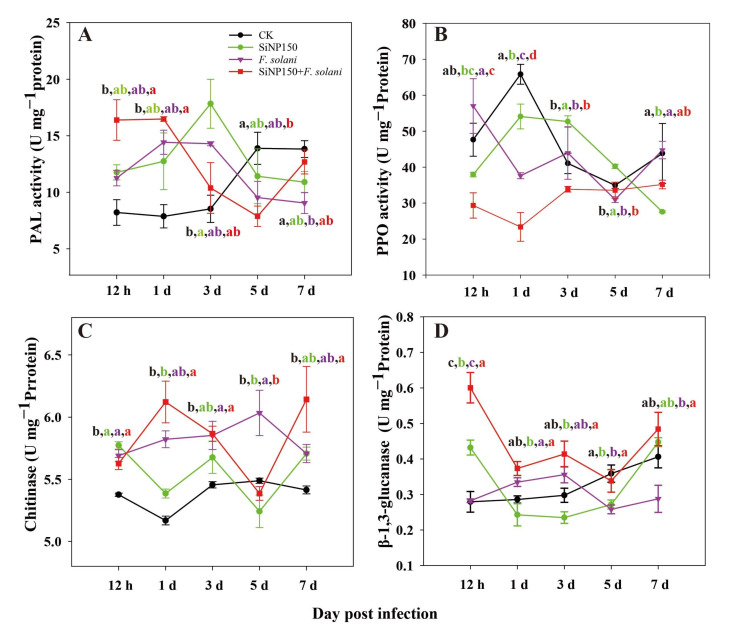
Effects of SiNPs and *F. solani* inoculation on the activities of (**A**) PAL, (**B**) PPO, (**C**) CHI, and (**D**) GLU in ginger rhizomes during postharvest storage. Results represent the mean ± standard deviation (SD). Different small letters in the figure show significant difference, the same letter indicates no difference (*p* < 0.05).

In the control group, the phenylalanine ammonia-lyase (PAL) activity increased gradually throughout the treatment (Figure 5A). In the SiNP150 treatment, the PAL activity increased before 3 days and then decreased in the following days, reaching the minimum (7.2 U mg^−1^ protein) on the 5th day (Figure 5A). In the *F. solani* treatment sample, the PAL activity firstly increased and then decreased. Compared with *F. solani* treatment alone, the SiNP150 + *F. solani* treatment increased the PAL activities at the early (0.5 days and 1 day) and later stages (7 days) but decreased it at 3 and 5 days (Figure 5A).

As shown in Figure 5B, in the CK and SiNP150 treatments, the PPO activity increased markedly within 1 day and then decreased during the later period of storage. Compared with *F. solani* treatment alone, the SiNP150 + *F. solani* treatments decreased the PPO activity throughout the experiment except for the 5th day.

As shown in Figure 5C, compared with CK, the other three treatments generally increased the chitinase (CHI) activity, except for the 5th day, where the SiNP150 and SiNP150 + *F. solani* treatments slightly decreased the CHI activity. Compared with *F. solani* treatment alone, the SiNP150 + *F. solani* treatment increased the CHI activity on the 1^st^ and 7th days but decreased it on the 5th day.

As indicated in Figure 5D, the β-1,3-glucanase (GLU) in CK and SiNP150 and GLU in *F. solani* and SiNP150 + *F. solani* largely showed similar fluctuating trends throughout the experiment, except for at day 0.5, in which the SiNP150 treatment inoculated with or without *F. solani* increased the GLU activity compared with CK and *F. solani* treatment alone. Specifically, SiNP150 + *F. solani*, *F. solani* treatment alone, and CK increased the activity of GLU compared with SiNP150, among which the activity of the SiNP150 + *F. solani* treatment was the highest, followed by *F. solani* treatment alone and CK. In this study, *F. solani* treatment alone increased the chitinase activity throughout the experiment and increased GLU activity at 1 and 3 days of treatment, which might result from pathogen induction (Figure 5C,D).

### 3.6. Effects of the SiNPs Treatment on Lignin, Total Flavonoid and Phenolics Contents

**Figure 6:**
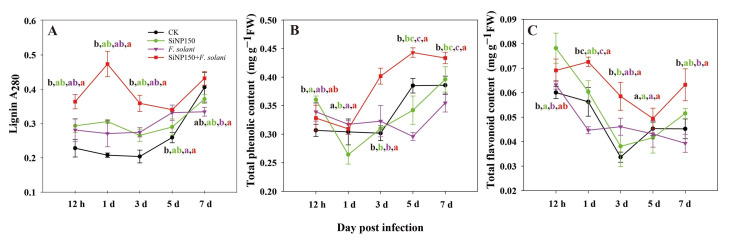
Effects of SiNPs and *F. solani* inoculation on the contents of (**A**) lignin, (**B**) total phenolic, and (**C**) total flavonoid contents in ginger rhizomes during postharvest storage. Results represent the mean ± standard error deviation (SD). Different small letters in the figure show significant difference, the same letter indicates no difference (*p* < 0.05).

As shown in Figure 6A, in the CK and SiNP150 treatments, the lignin content increased throughout the experiment. The lignin contents of the SiNP150- and *F. solani*-treated samples were higher than in CK before 5 days of treatment. In the SiNP150 + *F. solani* treatment sample, the lignin content increased dramatically at the initial storage stage (1 day), decreased sharply during the later storage period (3–5 days), and increased again on the 7th day. Compared with *F. solani* treatment alone, the SiNP150 + *F. solani* treatment increased the lignin content throughout the experiment, especially after 1 and 7 days of treatment, at which time points the lignin content increased by 75% and 27.3%, respectively.

In the CK, SiNP150, and SiNP150 + *F. solani* treatments, the total phenolic content presented an increasing trend after 1 day of treatment (Figure 6B). In the *F. solani* treatment alone, the total phenolic content generally decreased before 5 days of treatment, followed by an increase on the 7th day of treatment. Compared with *F. solani* treatment alone, the SiNP150 + *F. solani* treatments increased the total phenolic content by 1.3, 1.5, and 1.2 times after 3, 5, and 7 days of treatment.

As shown in Figure 6C, the total flavonoid content first decreased (before 3 days of treatment) and then increased (3–7 days of treatment). The total flavonoid content in the SiNP150 treatment showed a similar variation trend as the control but was higher than that of the control at most time points (except for the 7th day of treatment). Compared with *F. solani* treatment alone, the SiNP150 + *F. solani* treatment increased the total flavonoid content throughout the experiment by 9.3%, 62.4%, 26.9%, 12.8%, and 60.8% at each of the respective time points.

### 3.7. Effects of the SiNP150 Treatment on the Expression of Key Phenylpropanoid Pathway Genes Related to Lignin and Flavonoid

To explore the effect of SiNP150 on the expression levels of phenylpropanoid pathway-related genes, 5 PAL (PAL-1, PAL-2, PAL-3, PAL-4, and PAL-5), 2 C4H (cinnamate4-hydroxylase, C4H-1, and C4H-2), four 4CL (4-coumarate: CoA ligase, 4CL-1, 4CL-2, 4CL-3 and 4CL-4), 3 CHS (chalcone synthase, CHS-1, CHS-2, and CHS-3), 3 CCR (cinnamoyl CoA reductase, CCR-1, CCR-2, and CCR-3), and 3 CHI (chalcone isomerase, CHI-1, CHI, and CHI-3), 1 COMT (Caffeic acid-O-methyltransferase), 3 AQP (aquaporins, AQP-1, AQP-2, and AQP-3), and 4 SWEET genes (Sugars Will Eventually be Exported Transporter, SWEET-1, SWEET-2, SWEET-3 and SWEET-4) with relatively higher transcript levels within each gene family were selected based on our previous released RNA-seq data sets [2] to perform qRT-PCR analysis.

**Figure 7:**
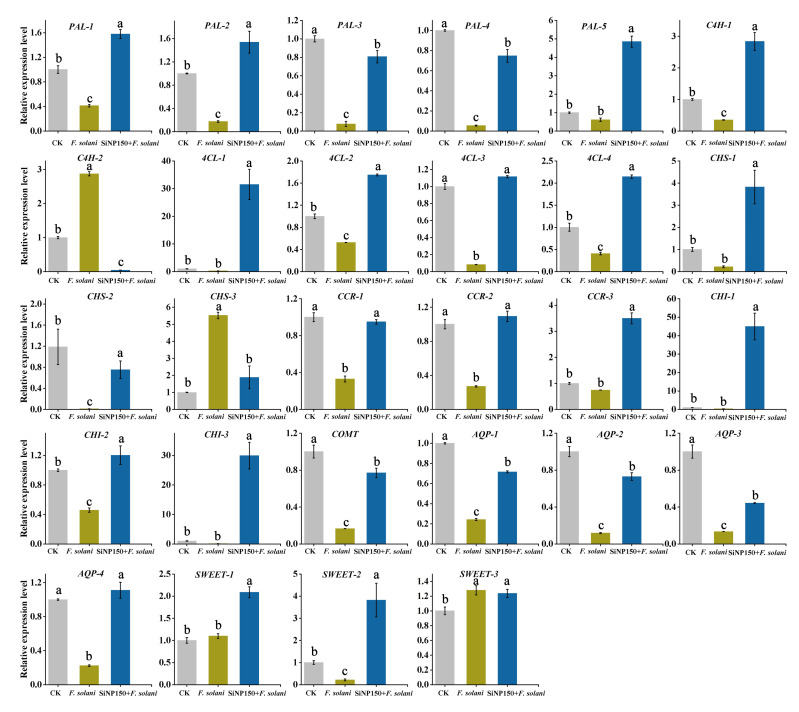
Effects of SiNPs and *F. solani* inoculation on the expression of genes related to the phenylpropanoid pathway, *SWEET* genes, and *AQPs*. Results represent the mean ± standard deviation (SD). Different small letters in the figure show significant difference, the same letter indicates no difference (*p* < 0.05).

Compared with control, *F. solani* treatment alone decreased the expression levels of 4 PAL genes, while SiNP150 + *F. solani* alleviated the decrease (Figure 7). To be specific, SiNP150 + *F. solani* increased the expression levels of PAL-1, PAL-2, PAL-3, PAL-4, and PAL-5 by 73.8%, 88.8%, 91.2%, 93.2%, 87.4%. *F. solani* treatment increased the expression levels of C4H-2, whereas it decreased C4H-1. In contrast, SiNP150 + *F. solani* decreased the expression levels of C4H-2 but increased C4H-1 compared with *F. solani* and CK. *F. solani* treatment alone decreased the expression levels of four 4CL genes, while SiNP150 + *F. solani* increased their expression levels by 96.8%, 42.5%, 9.9%, and 52.4%. Compared with the control, *F. solani* treatment alone decreased the expression levels of CHS-1 and CHS-2 and increased the expression levels of CHS-3. Compared with *F. solani* treatment alone, SiNP150 + *F. solani* increased the expression levels of CHS-1 and CHS-2 by 94.2% and 98.7%, respectively. SiNP150 + *F. solani* decreased the expression levels of CHS-3 as compared with *F. solani* treatment alone, but still higher than that of CK. The expression level of CCR, CHI, and CMOT genes was decreased by *F. solani* infection, while SiNP150 mitigated this decrease. SiNP150 + *F. solani* treatment increased the expression of CCR-2, CCR-3, and three CHI genes by 8.3%, 71.3%, 97.8%, 16.6%, 96.7% as compared with CK, and 75.2%, 78.8%, 99.2%, 62.5%, and 99.6% as compared with *F. solani*.

AQPs and SWEET proteins are important regulators of plant-pathogen interactions in higher plants. *F. solani* infection decreased the expression levels of 4 AQPs, while SiNP150 addition increased the expression levels of AQP-1, AQP-2, AQP-3, and AQP-4 by 2.9, 6.6, 3.4, and 5-times as compared with *F. solani* (Figure 7). *F. solani* infection with or without SiNP150 treatment increased the expression levels of SWEET-1 and SWEET-3. SiNP150 treatment further increased the expression levels of SWEET-1 and SWEET-2 as compared with *F. solani*.

## 4. Discussion

The postharvest decay of fruits and vegetables caused by pathogenic microorganisms needs to be controlled to reduce economic loss. Recently, nanomaterial treatments such as SiNPs have been investigated to reduce the application of synthetic fungicides for the control of postharvest rot in fruit and vegetables [6]. Our results showed that SiNP treatment had little significant inhibitory effect on the mycelial growth and spore germination of *F. solani* in vitro (Figure 1C,D), and different concentrations of SiNPs did not significantly alter the MDA content and SDH activity of the mycelium (Figure 1E,F). However, in some cases, it has been reported that sodium silicate (conventional bulk silicon) could directly inhibit spore germination and germ tube elongation [23,24]. For instance, research has found that sodium silicate could directly inhibit the spore germination and germ tube elongation of *Trichothecium roseum*, whereas silicon dioxide was ineffective [24]. Therefore, SiNPs may exhibit different physical and chemical properties than conventional bulk silicon, and the effect of SiNPs might be microorganism-dependent. Moreover, it may not directly influence the growth of pathogens such as *F. solani* but rather through other modes of action.

One of the mechanisms that enable SiNPs to improve plant disease resistance includes their deposition in the epidermal cell wall, which can form a mechanical barrier that hinders the penetration of fungal attachments [25,26]. In this study, the SiNP150 treatment decreased the mycelial growth of *F. solani* around the wounds (Figure 2A). SEM analysis showed that a white silica layer formed on the surface of the ginger epidermis, which may contribute to the inhibition of hyphae invading the cell (Figure 2B). Additionally, the particle sizes of the material used in this study were in the nanometer range, which could have made it possible for the chemical to penetrate into the exocarp and form deposits. These results suggest a mechanism similar to the hypothesis that Si-based compounds could conceivably form mechanical reinforcement to the invasion of fungal pathogens.

In addition to forming a mechanical barrier, Si may enhance plant defense against pathogens by activating the biochemical defense response. Studies have reported that sodium silicate treatment potentiated induced mitochondrial ROS accumulation, such as H_2_O_2_ and O_2_^−^, in pathogen-infected muskmelon fruits and wheat [7,25]. However, in this study, SiNP150 significantly reduced the O_2_^−^ and H_2_O_2_ levels after 7 days of *F. solani* treatment (Figure 3C,D). The inconsistent interaction of H_2_O_2_ and O_2_^−^ content may be caused by the differences in sampling times. In the present study, H_2_O_2_ and O_2_^−^ were detected at a later treatment period. H_2_O_2_ production is a defense response during the early biotrophic phase of the interaction, whereas higher concentrations of H_2_O_2_ at a later stage favor disease development [27]. Moreover, SiNPs have been reported to stimulate the activity of antioxidant enzymes, which might scavenge excessive ROS and protect the tissues from pathogen injury [28]. Similarly, enhanced defense enzyme activity, such as POD, and increased SOD, CAT, and APX activities after 1 and/or 3 days of SiNP150 treatment may have contributed to decreased O_2_^−^ and H_2_O_2_ levels in the later period of treatment (Figure 4).

Studies attribute the other ways by which Si deters pathogen invasion to the establishment of chemical impediments, such as (i) the activation of plant defense-related enzymes; and (ii) increased expression of genes related to plant defense mechanisms and encoding of chief enzymes in the production of phenylpropanoids [29]. Chitin and pectin are two main components of the fungal cell wall, which can be hydrolyzed by ‘pathogenesis-related proteins (PR)’ CHI and GLU produced by plant cells [30,31]. Conventional bulk silicon sodium has been reported to activate plant defenses in the early defense stage. Similarly, calcium silicate enhanced the resistance of banana roots to *F*. *oxysporum* by improving CHI, GLU, and PAL activities [32]. In this study, SiNP150 and SiNP150 + *F. solani* treatments increased the CHI, PAL, and β-1,3-glucanase activity at the onset of the experiment (12 h and/or 1 d), suggesting that silica nanoparticles may also be regarded as effective elicitor to activate plant defenses like conventional bulk silicon. In the present study, lower activity of CHI and GLU in the middle of the treatment (3–5 days) might be due to the improved resistance induced by SiNP150, which inhibited mycelial growth and penetration into cells (Figure 5C). Therefore, there is no need for plants to maintain a higher activity of CHI in a certain stage of treatment. Recent studies reported that silica nanoparticles and soluble Si(OH)_4_ can induce systemic acquired resistance in Arabidopsis plants against the bacterial pathogen *Pseudomonas syringae* [9]. These enzymes in the present study may partly contribute to the systemic acquired resistance induced by SiNP150 in ginger rhizomes.

Some studies in plants have reported the association of Si with lignin [33]. PAL is a key enzyme in the phenylpropanoid pathway and is closely related to the synthesis of secondary metabolites (e.g., lignin and flavonoids) that play important roles in the pathogen invasion resistance process [34,35]. Therefore, the increase in the activity of PAL at the early (0.5–1 days) and later stages of inoculation (7 days) (Figure 5A) may also contribute to the increased lignin content in the ginger rhizomes due to SiNP supplementation, which can effectively inhibit the expansion of pathogens. Similarly, SiNPs enhanced PAL expression and lignification in the leaves and roots of oat seedlings [36].

Secondary metabolites derived through the phenylpropanoid pathway, such as phenols and flavonoids, have been implicated in Si-induced resistance against fungal pathogens [37]. For instance, Si-induced enhanced accumulation of phenols and flavonoids contributed to enhanced resistance to powdery mildew in bitter gourd [38]. Similarly, in this study, SiNP150 treatment increased the phenol and flavonoid content in pathogen-inoculated ginger rhizomes throughout the experiment. At the same time, in un-inoculated plants, SiNP150 enhanced phenol and flavonoid contents at the early stage of treatment (Figure 6). The changes in phenol and flavonoid content seem to be inconsistent with the fluctuations in PAL in the middle stage of the treatment. There could be several reasons for this difference. Exquisite regulatory mechanisms at multiple levels control the enzymatic activity and transcription of PALs. Moreover, PAL and phenylpropanoid biosynthetic activity appear to be metabolically regulated by particular biosynthetic intermediates or chemical signals (metabolite feedback regulation) [39].

Furthermore, phenylpropanoid pathway-related genes, including 5 *PAL*, 2 *C4H*, 4 *4CL*, 3 *CHS*, 3 *CCR*, 3 *CHI*, and 1 *COMT* with relatively higher transcript levels within each gene family were selected to explore the possible regulating effect of SiNPs in lignin and flavonoids metabolisms in the inoculated ginger rhizome. The increased expression levels of 5 *PAL*, 1 *C4H*, 4 *4CL*, 2 *CHS*, 3 *CCR*, 3 *CHI*, and 1 *COMT* transcripts with SiNP150 addition compared with *F. solani* treatment alone (Figure 7) are consistent with SiNPs-induced accumulation of phenolics, flavonoids, and lignin. Our results indicated that Si triggered stronger defense responses in pathogen inoculated samples through stimulating the activities of POD, defense enzymes such as PAL, GLU, and phenylpropanoid pathway-related genes expression.

To further explore the possible transcriptional regulation associated with the increase in plant resistance to disease by Si, the expression of several AQPs and SWEETs was studied based on our previous transcriptome studies. AQPs are membrane channel proteins primarily associated with water and small solute transport across cell membranes [40]. The SWEET proteins are a novel family of sugar transporters that mediate sugar translocation, signal transduction, plant-pathogen interactions, and stress tolerance [41]. Increasing evidence suggests that AQPs and SWEET play important roles in plant-pathogen interactions [42]. In this study, SiNP150 + *F. solani* treatment alleviated the decrease of four *AQP* genes (PIPs) under stress conditions, which may contribute to immune responses in ginger rhizomes. Considering that small molecules (e.g., nitric oxide, silica, and H_2_O_2_) are involved in a variety of metabolic processes and functions associated with plant immunity are transported across the membrane via AQP channels, a more comprehensive understanding of *AQPs* in ginger immunity and pathogen pathogenicity during their interaction is needed.

Most studies support that pathogen attack upregulates *SWEET* genes. At the same time, a few reports suggest that pathogen attack not only upregulates *SWEET* genes but also downregulates or negatively regulates *SWEET* genes [42]. In this study, *F. solani* infection upregulated *SWEET-1* and *SWEET-3* but decreased *SWEET-2* expression. This downregulation of *SWEET-2* might be due to the interruption of sugar signaling pathways [43]. It is difficult to identify a concerted pattern of *SWEET* expression in response to SiNP150 treatment. The SiNP150 + *F. solani* treatment appeared to further increase the expression levels of *SWEET-1* and *SWEET-2* compared with CK and *F. solani* treatment, a finding that needs to be further confirmed in more *SWEET* gene family members. In addition, a further in-depth study is required to explore the differential expression of *SWEET* genes during pathogen attack to better understand the physiology of plant–pathogen interactions.

## 5. Conclusions

**Figure 8:**
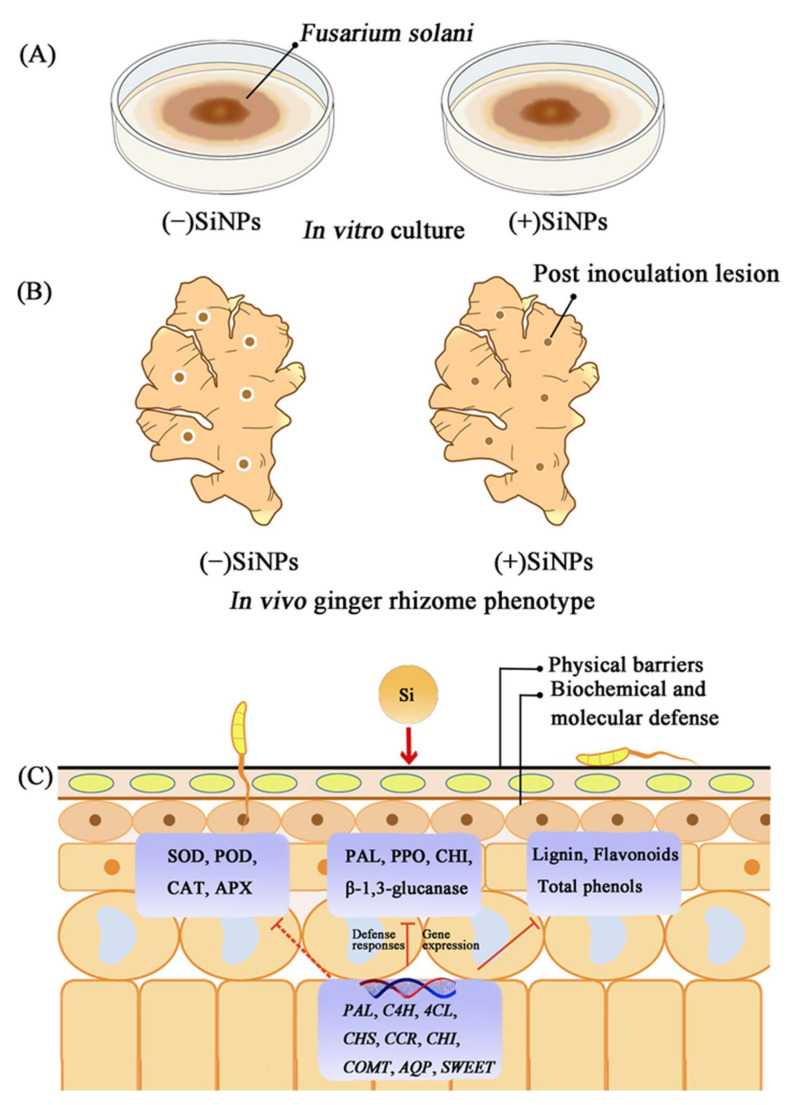
Mechanism diagram of SiNPs-induced enhancement of postharvest disease resistance of ginger rhizomes. (**A**) In vitro antifungal activity of SiNPs. (**B**) Hypothetical ginger rhizome phenotypes showing suppression of decay development by Si application in vivo. (**C**) Physical, biochemical, and molecular defense mechanisms of SiNPs -induced resistance to postharvest diseases caused by pathogenic fungi. Generally, SiNPs deposition on the surface of ginger rhizomes prevents pathogenic fungi from penetrating epidermal cells. Meanwhile, SiNPs induced defense response involves the accumulation of secondary metabolites such as phenols, flavonoids, and lignins, as well as an increase in the activity of antioxidant enzymes such as SOD, POD, CAT, APX, and defense enzymes such as PAL, PPO, CHI, β-1,3-glucanase, which may be fine-tuned by the regulation of related genes. (*PAL*, Phenylalanine ammonialyase; *C4H*, cinnamate4-hydroxylase; *4CL*, 4-coumarate: CoA ligase; *CHS*, chalcone synthase; *CCR*, cinnamoyl CoA reductase; *CHI*, chalcone isomerase; *COMT*, Caffeic acid-O-methyltransferase; *AQP*, aquaporins; *SWEET*).

The silica dioxide nanoparticles reduced *Fusarium solani* caused postharvest decay through physical and biochemical defense mechanisms. The application of SiNPs maintained the antioxidant defense system of ginger at a high level in response to long-term pathogenic infection and hence reduced disease indices. Moreover, the nanoparticles induced defense-related enzyme activity and phenylpropanoid pathway-related gene expression in ginger rhizomes. Therefore, SiNPs can be used as an alternative tool to chemical fungicides to promote the defense response in postharvest ginger, thus efficiently and environmentally friendly managing rhizome rot disease (Figure 8).

## Data Availability

Data are real and effective.

## Supplementary Materials

The following supporting information can be downloaded at: https://www.mdpi.com/article/10.3390/nano12091418/s1, Figure S1. The SEM (A), TEM (B) micrographs and FTIR spectra (C) of silica nanoparticles used in this study, Table S1. The primers used for qRT-PCR, Table S2. Abbreviations list in this study.
