# Supplementary material for: Silica Nanoparticles Enhance the Disease Resistance of Ginger to Rhizome Rot during Postharvest Storage
Source: Nanomaterials (Basel). 2022 Apr 21;12(9):1418. doi: 10.3390/nano12091418 (PMC9099806; doi:10.3390/nano12091418)
Supplement: Supplementary file 1 [file nanomaterials-12-01418-s001.zip › nanomaterials-1647217-supplementary.pdf]

nanomaterials

MDPI

Supplementary Materials

Silica Nanoparticles Enhance the Disease Resistance of Ginger to Rhizome Rot during Postharvest Storage

Jie Zhou [1,†], Xuli Liu [1,†], Chong Sun [1,2], Gang Li [1], Peihua Yang [1], Qie Jia [1], Xiaodong Cai [1], Yiqing Liu [1,*], Junliang Yin [1,*] and Yongxing Zhu [1,*]

[1] Spice Crops Research Institute, College of Horticulture and Gardening, Yangtze University, Jingzhou 434025, China; zj188719@163.com (J.Z.); liuxuli33@163.com (X.L.); zbgqsc1987@163.com (C.S.); lg13733590933@163.com (G.L.); Yph919701@163.com (P.Y.); jiaqie020@163.com (Q.J.); caixiao.dong@163.com (X.C.)

[2] Special Plants Institute, College of Landscape Architecture and Life Science, Chongqing University of Arts and Sciences, Chongqing 402160, China

* Correspondence: xbnlzyx@163.com (Y.Z.); w.yinzi@163.com (J.Y.); liung906@163.com (Y.L.)

† These authors contributed equally to this work.

The morphology of SiNPs was characterized using SEM (Figure S1A) and TEM (Figure S1B) analyses which reveal almost spherical nanoparticles. The FTIR spectra (Figure S1C) displayed the broad peaks detected at 1105.33 (corresponding to the Si-O-Si) and 470.95 cm^{-1} (corresponding to the Si-O band) ranges. These results revealed that the silica particles were amorphous in nature and on a nanoscale. No other impurities were detected. Therefore, the prepared SiNPs has been used in this study due to high stability and purity.

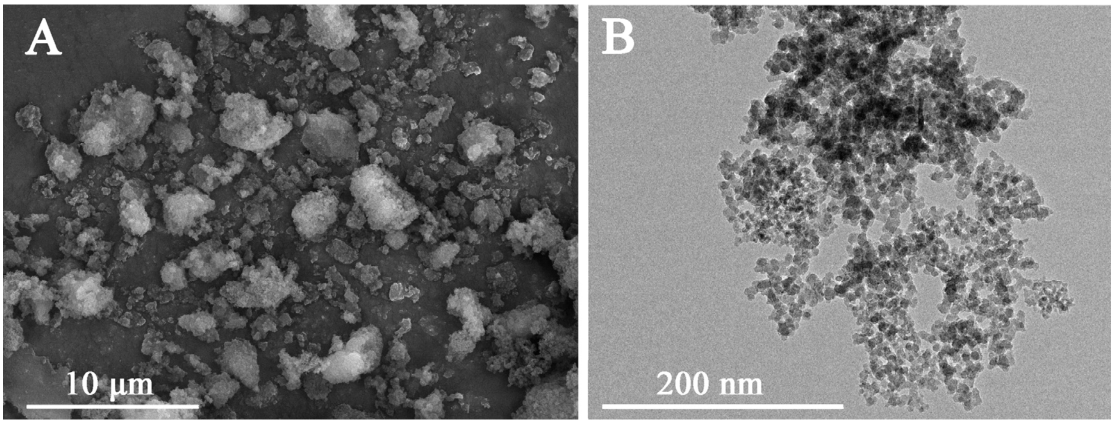

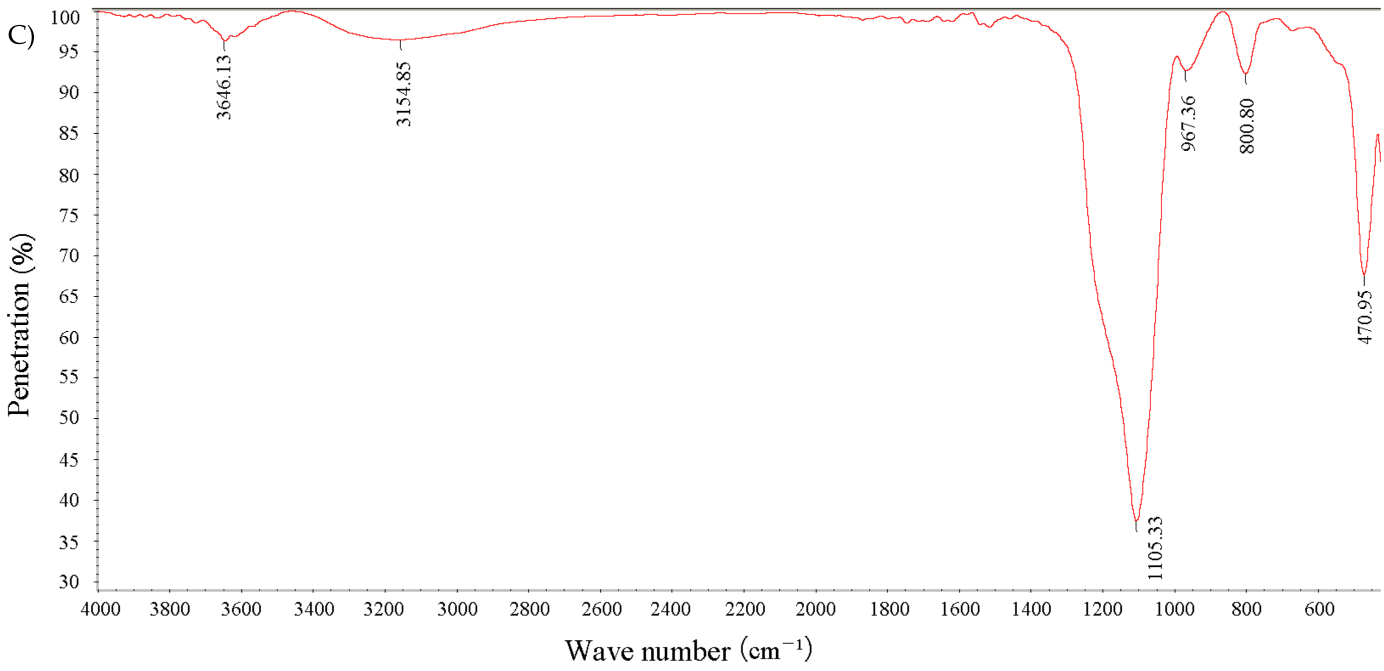

Figure S1. The SEM (**A**), TEM (**B**) micrographs and FTIR spectra (**C**) of silica nanoparticles used in this study.

Table S1. The primers used for qRT-PCR.

**Gene**		Annotaion	Primer Sequences (Forward/Reverse Primer)	Amplicon Length (bp)
**PAL-1**	Maker00078705	phenylalanine ammonia-lyase-like	F 5′-GAGCAGCACAACCAGGAC-3′	192
PAL-1	Maker00078705	phenylalanine ammonia-lyase-like	R 5′-CACCCGCTTGGACACTT-3′	
PAL-2	Maker00014343	phenylalanine ammonia-lyase-like	F 5′-CATCCCGCACGGCTTCTTC-3′	324
PAL-2	Maker00014343	phenylalanine ammonia-lyase-like	R 5′-CGGTCCTGCTTCGGCTTT-3′	
PAL-3	Maker00014397	phenylalanine ammonia-lyase-like	F 5′-GGCGACAAGGAGAAGGACG-3′	296
PAL-3	Maker00014397	phenylalanine ammonia-lyase-like	R 5′-GCGAGCAAAGGATCAACGAG-3′	
PAL-4	Maker00014533	phenylalanine ammonia-lyase-like	F 5′-GGCGACAAGGAGAAGGACG-3′	303
PAL-4	Maker00014533	phenylalanine ammonia-lyase-like	R 5′-CAAGCAATCGAGCAAGGGAT-3′	
PAL-5	Maker00028690	phenylalanine ammonia-lyase-like	F 5′-GTGAACGGCACAGCAGTCG-3′	63
PAL-5	Maker00028690	phenylalanine ammonia-lyase-like	R 5′-GAGGATATTGGCGTCGTAGAGG-3′	
C4H-1	Maker00064356	trans-cinnamate 4-monooxygenase-like	F 5′-TCAACCACCGAAACCTCAC-3′	339
C4H-1	Maker00064356	trans-cinnamate 4-monooxygenase-like	R 5′-TGGCGTCCTTCTTCACATC-3′	
C4H-2	Maker00071635	trans-cinnamate 4-monooxygenase-like	F 5′-TGGAGGCAAACGGCAATG-3′	138
C4H-2	Maker00071635	trans-cinnamate 4-monooxygenase-like	R 5′-GCGGCGGTAACAAATGGA-3′	
4CL-1	Maker00028796	4-coumarate--CoA ligase 2-like	F 5′-AACTCCGTCCTCCTTTGCTG-3′	154
4CL-1	Maker00028796	4-coumarate--CoA ligase 2-like	R 5′-GTGGGCTCTTCACGAACTCC-3′	
4CL-2	Maker00039000	4-coumarate--CoA ligase-like 1	F 5′-GACAACTGGAGCGAGCAAG-3′	165
	Maker00039000	4-coumarate--CoA ligase-	R 5′-TGAGCGTAGGTAATCACAGACAA-3′	

4CL-2		like 1		
4CL-3	Maker00051484	probable 4-coumarate--CoA ligase 3	F 5´-CTGAAGCAACGAGGAACACG-3´	173
4CL-3	Maker00051484	probable 4-coumarate--CoA ligase 3	R 5´-TGGGTGAGCGACGAGCAA-3´	
4CL-4	Maker00019269	probable 4-coumarate--CoA ligase 3	F 5´-TAAGGGCGTGATGCTGACC -3´	135
4CL-4	Maker00019269	probable 4-coumarate--CoA ligase 3	R 5´-AGGGAGTAGATGTGGAAGAGGG -3´	
CHS-1	Maker00037685	chalcone synthase	F 5´-GACTGAGGAGATGCTGAGGGAG-3´	233
CHS-1	Maker00037685	chalcone synthase	R 5´-GGCCGAGGAGCTTGGTGA-3´	
CHS-2	Maker00037732	chalcone synthase	F 5´-AGATGCTGAGTGAGAACCCG-3´	224
CHS-2	Maker00037732	chalcone synthase	R 5´-GCCGAGGAGCTTGGTGA-3´	
CHS-3	Maker00013900	chalcone synthase 2	F 5´-GGCGAGGGTTTCAACTGGG -3´	64
CHS-3	Maker00013900	chalcone synthase 2	R 5´-CGACGGTCTCGACGGTGATT-3´	
CCR-1	Maker00013349	cinnamoyl-CoA reductase 1	F 5´-CAGCGGTTTCATCGGTTC-3´	139
CCR-1	Maker00013349	cinnamoyl-CoA reductase 1	R 5´-GCGAAGGCGATTGGAG-3´	
CCR-2	Maker00076587	probable 4-coumarate--CoA ligase 2	F 5´-CACCGCCAATCCATTCTCC-3´	146
CCR-2	Maker00076587	probable 4-coumarate--CoA ligase 2	R 5´-CCACCGTCAAACCCTCACC-3´	
CCR-3	Maker00037604	cinnamoyl-CoA reductase 1	F 5´-CGGGCATTGTTTGCGTCAC-3´	166
CCR-3	Maker00037604	cinnamoyl-CoA reductase 1	R 5´-AGGATAAGGCGGTCGGAGG-3´	
CHI-1	Maker00019194	vacuolar-sorting receptor 6	F 5´-TTACCAGGGATAACCAGAAAT-3´	57
CHI-1	Maker00019194	vacuolar-sorting receptor 6	R 5´-GGCAGCCAGTCAGTTGAG-3´	
CHI-2	Maker00008848	vacuolar-sorting receptor 1	F 5´-GACGACAAGGCGGACTATC-3´	315
CHI-2	Maker00008848	vacuolar-sorting receptor 1	R 5´-GGCTTCAGGGCAATACCA-3´	
CHI-3	Maker00005583	vacuolar-sorting receptor 6	F 5´-TACCAGGGATAACCAGACATTC-3´	151
CHI-3	Maker00005583	vacuolar-sorting receptor 6	R 5´-GCAGGAGCATTCAGGACAA-3´	
COMT-1	Maker00033993	hypothetical protein B296_00011742	F 5´-AGCATCTCCTTCCCAAATAC-3´	263
COMT-1	Maker00033993	hypothetical protein B296_00011742	R 5´-GCACAGCTCGTCACTCC-3´	
AQP-1	Maker00015089	probable aquaporin PIP1-2	F 5´-GGGAAGGAGGAGGATGTGA-3´	145
AQP-1	Maker00015089	probable aquaporin PIP1-2	R 5´-GGTAGAAGGACCAGGAACTGA-3´	
AQP-2	Maker00037662	aquaporin PIP2	F 5´-AAGGAGGTTAGCGTGGAGG-3´	171
AQP-2	Maker00037662	aquaporin PIP2	R 5´-GATGACGGTAGCGATGGTGA-3´	
AQP-3	Maker00052469	aquaporin PIP2	F 5´-CGAAGGAGGTTAGTGAGGAGG-3´	219
AQP-3	Maker00052469	aquaporin PIP2	R 5´-CGCCGTCGCATTGGTT-3´	
AQP-4	Maker00037595	plasma membrane intrinsic protein 1	F 5´- GTACGAGAACAATGGAGGAGG-3´	108
AQP-4	Maker00037595	plasma membrane intrinsic protein 1	R 5´-AAGACAGTGTAGACGAGGATGAA-3´	
SWEET-1	Maker00022952	bidirectional sugar transporter SWEET4-like	F 5´-ATGACAATCTCAGCCGAAAC-3´	178
SWEET-1	Maker00022952	bidirectional sugar transporter SWEET4-like	R 5´-GCACCCACAGCAAGCAG-3´	
SWEET-2	Maker00016728	bidirectional sugar transporter SWEET2a-like isoform X2	F 5´-TCCCTTGCCACATTCTTG-3´	151
SWEET-2	Maker00016728	bidirectional sugar transporter SWEET2a-like iso-	R 5´-CCTCTTCGGATTTCCTGCT-3´	

		form X2	
SWEET-3	Maker00069608	bidirectional sugar transporter SWEET2a-like	F 5´-GCAATGTCTTCGCCTTCG-3´
SWEET-3	Maker00069608	bidirectional sugar transporter SWEET2a-like	R 5´-TTGTCGTGTCGGGTGGTC-3´

Table S2. Abbreviations list in this study.

Full name	Abbreviation
Silica nanoparticles	SiNPs
Silicon	Si
Reactive oxygen species	ROS
Potato dextrose agar	PDA
Thiobarbituric acid	TCA
Diaminobenzidine	DAB
Nitroblue tetrazolium	NBT
Scanning electron microscopy	SEM
Hydrogen peroxide	H_2O_2
Superoxide anion	O_2^-
Malondialdehyde	MDA
Peroxidase	POD
Superoxide dismutase	SOD
Catalase	CAT
Ascorbate peroxidase	APX
Phenylalanine ammonia-lyase	PAL
Chitinase	CHI
β-1,3-glucosidase	GLU
Polyphenol oxidase	PPO
Cinnamate4-hydroxylase	C4H
4-coumarate: CoA ligase	4CL
Chalcone synthase	CHS
Cinnamoyl CoA reductase	CCR
Chalcone isomerase	CHI
Caffeic acid-O-methyltransferase	COMT
Aquaporins	AQP
Sugars Will Eventually be Exported Transporter	SWEET
Systemic acquired resistance	SAR
